# Selective Detection of Alkaline Phosphatase Activity in Environmental Water Samples by Copper Nanoclusters Doped Lanthanide Coordination Polymer Nanocomposites as the Ratiometric Fluorescent Probe

**DOI:** 10.3390/bios12060372

**Published:** 2022-05-28

**Authors:** Xin Li, Xiaoling Wang, Wei Guo, Yunfei Wang, Qing Hua, Feiyan Tang, Feng Luan, Chunyuan Tian, Xuming Zhuang, Lijun Zhao

**Affiliations:** 1 College of Chemistry and Chemical Engineering, Yantai University, Yantai 264005, China; lixinxin970519@163.com (X.L.); wxl18354380401@163.com (X.W.); yunfeiw0712@163.com (Y.W.); 13844630958@163.com (Q.H.); tangfeiyan0807@163.com (F.T.); luanf@ytu.edu.cn (F.L.); cytian@ytu.edu.cn (C.T.); 2 Shandong Dyne Marine Biopharmaceutical Co., Ltd., Weihai 264300, China

**Keywords:** alkaline phosphatase, copper nanoclusters, infinite coordination polymer, ratiometric, fluorescent probe

## Abstract

In this paper, a novel, accurate, sensitive and rapid ratiometric fluorescent sensor was fabricated using a copper nanoclusters@infinite coordination polymer (ICP), specifically, terbium ion-guanosine 5’-disodium (Cu NCs@Tb-GMP) nanocomposites as the ratiometric fluorescent probe, to detect alkaline phosphatase (ALP) in water. The fluorescence probe was characterized by scanning electron microscopy, transmission electron microscopy, X-ray photoelectron spectroscopy, and Fourier transform infrared spectroscopy. The experimental results showed that, compared with Tb-GMP fluorescent sensors, Cu ratiometric fluorescent sensors based on NCs encapsulated in Tb-GMP had fewer experimental errors and fewer false-positive signals and were more conducive to the sensitive and accurate detection of ALP. In addition, the developed fluorescent probe had good fluorescence intensity, selectivity, repeatability and stability. Under optimized conditions, the ratiometric fluorescent sensor detected ALP in the range of 0.002–2 U mL^−1^ (R^2^ = 0.9950) with a limit of detection of 0.002 U mL^−1^, and the recovery of ALP from water samples was less than 108.2%. These satisfying results proved that the ratiometric fluorescent probe has good application prospects and provides a new method for the detection of ALP in real water samples.

## 1. Introduction

In recent years, water eutrophication [1,2], which is caused by the excessive enrichment of nutrients such as nitrogen and phosphorus in water, has attracted increasing attention from society. Accumulating evidence suggests that alkaline phosphatase (ALP) not only provides available phosphorus to organisms in water but also plays a key role in the phosphorus cycle in water [3,4,5]. The ALP can be used as the index of phosphorus deficiency; the activity of ALP increases by 25 times when phosphorus deficiency occurs [6]. Meanwhile, ALP is an essential enzyme in phosphate metabolism because it can effectively catalyze hydrolysis or the transphosphorylation of phosphate. Therefore, accurately detecting the concentration of ALP in water, which is more important than other indicators used to evaluate water quality, is of great significance for ecological environments and human production activities [7,8,9]. To date, a variety of methods for detecting ALP, such as colorimetric [10], electrochemical [11], surface-enhanced Raman dispersion [12], and molecular fluorescence methods [13], have been reported. Among these methods, molecular fluorescence has attracted extensive attention due to its advantages of fast detection speed, small sample quantity and high sensitivity.

In the molecular fluorescence method, ratiometric fluorescent sensors have been widely used for the past few years because of their high selectivity and accuracy [14,15]. This method mainly analyzes the change in the ratios of the signal intensities at different fluorescence wavelengths, which can effectively reduce the error and false-positive signals in the experiment [16]. In recent years, the construction of ratiometric fluorescent sensors has attracted the attention of many researchers. Loas and his colleagues developed a Cu-based ratiometric fluorescent sensor for nitric oxide detection that operated on the energy transfer between hydroxycoumarin and luciferin chromophores [17]. Wang and coworkers designed a dual-emission ratiometric fluorescent sensor to detect folic acid by doping ZnS quantum dots with Cu^2+^ and Mn^2+^ ions [18]. Ye and his workmates developed a ratiometric fluorescent platform for the amplification of kanamycin without an enzyme signal to enable the detection of antibiotics [19]. Although many nanocomposites have been used in ratiometric fluorescent sensors, the development of a novel kind of nanocomposite with excellent performance is still worth studying.

To date, an infinite coordination polymer (ICP) nanocomposite based on terbium ions (Tb^3+^) and guanosine 5’-disodium (GMP) has been developed [20]. Compared with other coordination polymers, ICPs have obvious advantages, such as high structural flexibility [21], a good guest envelope, and a fast response to external stimuli [22,23,24]. Meanwhile, copper nanoclusters (Cu NCs) have gradually become a research and application hotspot in the fields of biological analysis and environmental monitoring with broad prospects because of their good biocompatibility, low biotoxicity, good photostability and low cost [25,26,27]. However, Cu NCs also have disadvantages such as easy oxidation and aggregation in the preparation process [25]. To overcome these difficulties, researchers have proposed many different solutions. Some researchers have used different materials to synthesize Cu NCs [28,29,30], whereas others have used ZIFs and other materials to encapsulate Cu NCs to form nanocomposites [31,32,33].

**Scheme 1:**
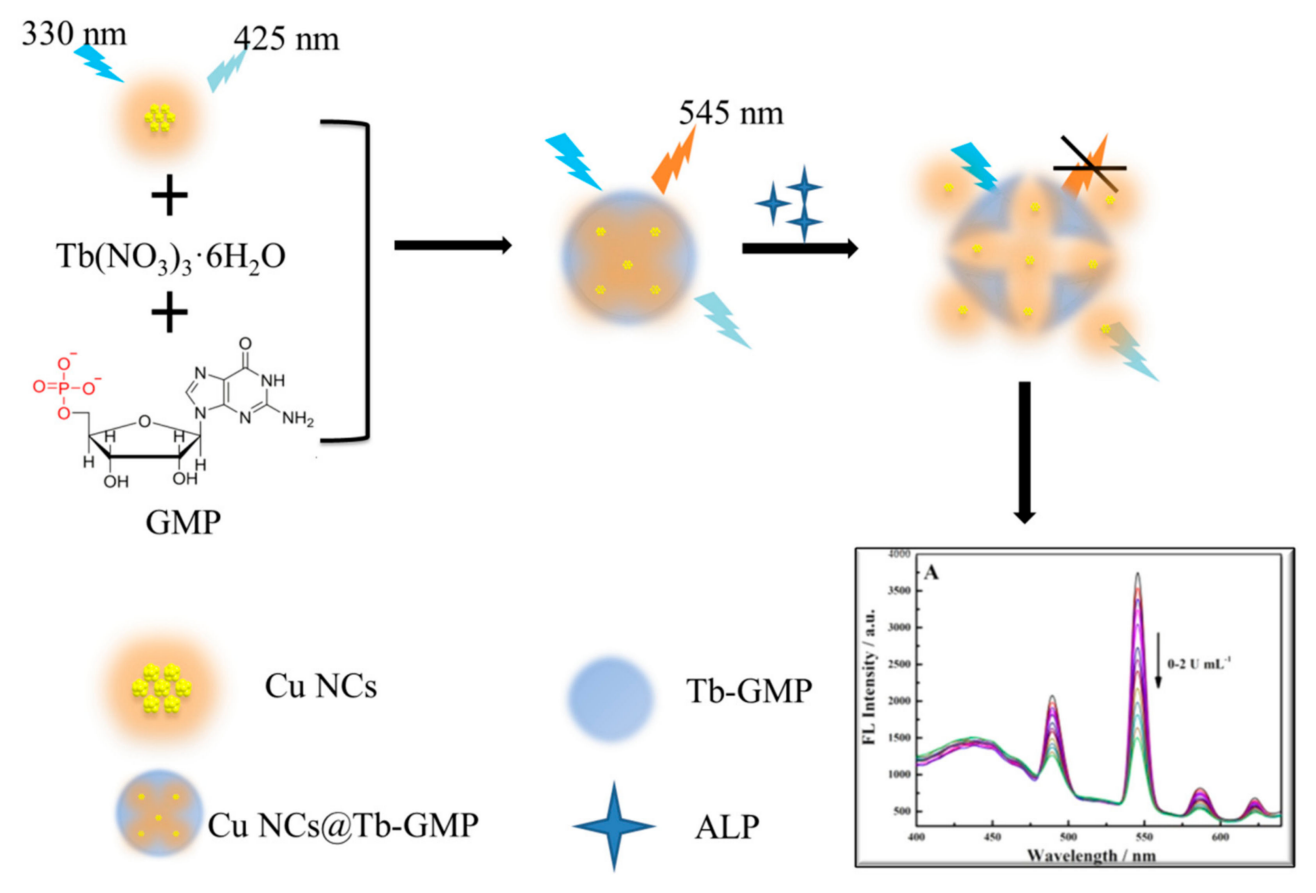
A schematic of the mechanism of Cu NCs@Tb-GMP for ALP detection.

In this paper, a ratiometric fluorescent probe named Cu NCs@Tb-GMP was successfully prepared and could detect ALP quickly. On the one hand, Cu NCs could be used to sensitize Tb-GMP to enhance its fluorescence intensity; on the other hand, using Cu NCs as an internal standard [34] could effectively correct the error and improve the accuracy of the experiment. The mechanism is shown in Scheme 1. In the presence of ALP, the phosphate group in GMP could be hydrolyzed by ALP, leading to the destruction of the Tb-GMP network, and the characteristic emission intensity of Tb-GMP at 545 nm was significantly reduced. At the same time, due to the hydrolysis of the polymer network, Cu NCs were released into the solution, resulting in a slight but almost negligible increase in their fluorescence at 425 nm [34,35]. Thus, the ratio of fluorescent intensity at 545 nm and 425 nm has been choosing as the ratiometric fluorescent parameter value. After a series of characterizations and condition optimization, the Cu NCs@Tb-GMP probe was successfully applied to the detection of ALP in environmental water samples under the optimal conditions. According to the experimental results, compared with other methods, the constructed molecular fluorescent probe had the advantages of higher stability, better sensitivity and excellent anti-interference ability. At the same time, the preparation method of the synthesized fluorescent probe was simple and the cost was relatively low; therefore, it has wide application prospects in the protection of ecological environmental systems and human health.

## 2. Materials and Methods

### 2.1. Materials

GMP, terbium nitrate hexahydrate (Tb(NO_3_)_3_ · 6H_2_O), n-2-hydroxyethylpiperazine- n’-2-ethanesulfonic acid (HEPES), ALP, glucose dehydrogenase (GDH), thrombin, glucose oxidase (GOx) and horseradish peroxidase (HRP) were purchased from Shanghai Aladdin Biochemical Technology Co., Ltd. (Shanghai, China). Copper sulfate pentahydrate (CuSO_4_ · 5H_2_O), ethylenediamine (EDA) and L-ascorbic acid (AA) were obtained from Sinopharm Chemical Reagent Co., Ltd. (Tianjin, China). Phosphate buffer solution (PBS, 0.1 M, pH = 7.4) was prepared from a standard mixture of sodium dihydrogen phosphate and dipotassium hydrogen phosphate. All the solutions involved in these experiments were prepared with ultrapure water (18.25 MΩ cm).

### 2.2. Apparatus

Scanning electron microscopy (SEM) images and transmission electron microscopy (TEM) images were obtained on JSM-7900F and JEM-2100 (200 kV) instruments, respectively (JEOL Ltd., Tokyo, Japan). High-resolution transmission electron microscopy (HR-TEM) images were obtained on a JEM-2100F transmission electron microscope at an accelerating voltage of 200 kV (JEOL Ltd., Tokyo, Japan). X-ray photoelectron spectroscopy (XPS) measurements were performed on a Thermo ESCALAB-250 instrument (Thermo Fisher Scientific, Waltham, MA, USA). The fluorescence emission spectra were obtained on an F-4700 fluorescence spectrophotometer (HITACHI, Tokyo, Japan). A Nicolet 5700 Fourier transform infrared (FT-IR) spectrometer (Thermo Electron Corporation, Waltham, MA, USA) was used to obtain FT-IR spectra. Ultraviolet-visible absorption spectroscopy (UV-vis) was performed with an A560 ultraviolet-visible spectrometer (AOE Instruments, Shanghai, China) at wavelength intervals of 2 nm.

### 2.3. Synthesis of Nanocomposites

#### 2.3.1. Synthesis of Cu NCs

Cu NCs were prepared according to a simple approach reported previously [25]. The specific steps were as follows: first, CuSO_4_ · 5H_2_O (0.08 mM), EDA (1.36 mM) and AA (0.800 mM) were dissolved in 24 mL of ultrapure water and adjusted with 1 M NaOH to keep the pH at approximately 4.5. Next, the solution obtained above was heated to 37 °C and reacted at a constant temperature for 30 min. Then, the solution was cooled to room temperature, transferred to a centrifuge tube and centrifuged at 4000 rpm for 10 min. After washing 3 times, the supernatant was removed and the precipitate was dispersed in ultrapure water to obtain the required Cu NCs, which were stored at 4 °C for further experiments.

#### 2.3.2. Synthesis of Tb-GMP and the Cu NCs@Tb-GMP Ratiometric Fluorescent Probe

According to reference [20], Tb-GMP and Cu NCs@Tb-GMP were obtained. The concentrations of Tb(NO_3_)_3_ · 6H_2_O and the GMP solution were both 10 mM. When the two solutions were mixed in equal volumes at room temperature, a white precipitate was obtained, washed with ultrapure water and centrifuged at 5000 rpm for 10 min. In this way, Tb-GMP was successfully synthesized. Cu NCs@Tb-GMP was obtained by a similar method. For the synthesis of the Cu NCs@Tb-GMP ratiometric fluorescent probe, the above synthesis steps were changed slightly to ensure encapsulation of Cu NCs in the Tb-GMP. The only difference was that Cu NCs were added to the Tb(NO_3_)_3_ · 6H_2_O solution during the Tb-GMP synthesis process to fully encapsulate Cu NCs in Tb-GMP.

### 2.4. Construction of the Ratiometric Fluorescent Sensor

A series of experiments was designed to develop ratiometric fluorescent sensors. First, an aqueous solution of ALP with an activity of 10 U mL^−1^ was prepared. In addition, a certain volume of the ALP aqueous solution was successively added to the Cu NCs@Tb-GMP ratiometric fluorescent probe, which resulted in an ALP activity in the Cu NCs@Tb-GMP aqueous solution of 0–2 U mL^−1^. Then, the mixed solution was placed in a thermostatic water bath at 37 °C for 30 min, ensuring full reaction. A fluorescence spectrophotometer was selected as the detection method. In this way, a ratiometric fluorescent sensor that could achieve sensitive detection of ALP in aqueous solutions was successfully fabricated.

### 2.5. Fluorescence Assay of ALP in Real Samples

Different samples were obtained from local river in Yantai City (water samples were taken from the same river in upper, middle and lower reach area) to evaluate the performance of the proposed method. First, the sample through a 0.45 μm mem-143 brane was filtered for further use. Next, the sample was diluted 10 times with PBS (0.1 M, pH = 7.4), which was stored at −20 °C for further use.

## 3. Results and Discussion

### 3.1. Characterization of the Cu NCs, Tb-GMP and Cu NCs@Tb-GMP

**Figure 1:**
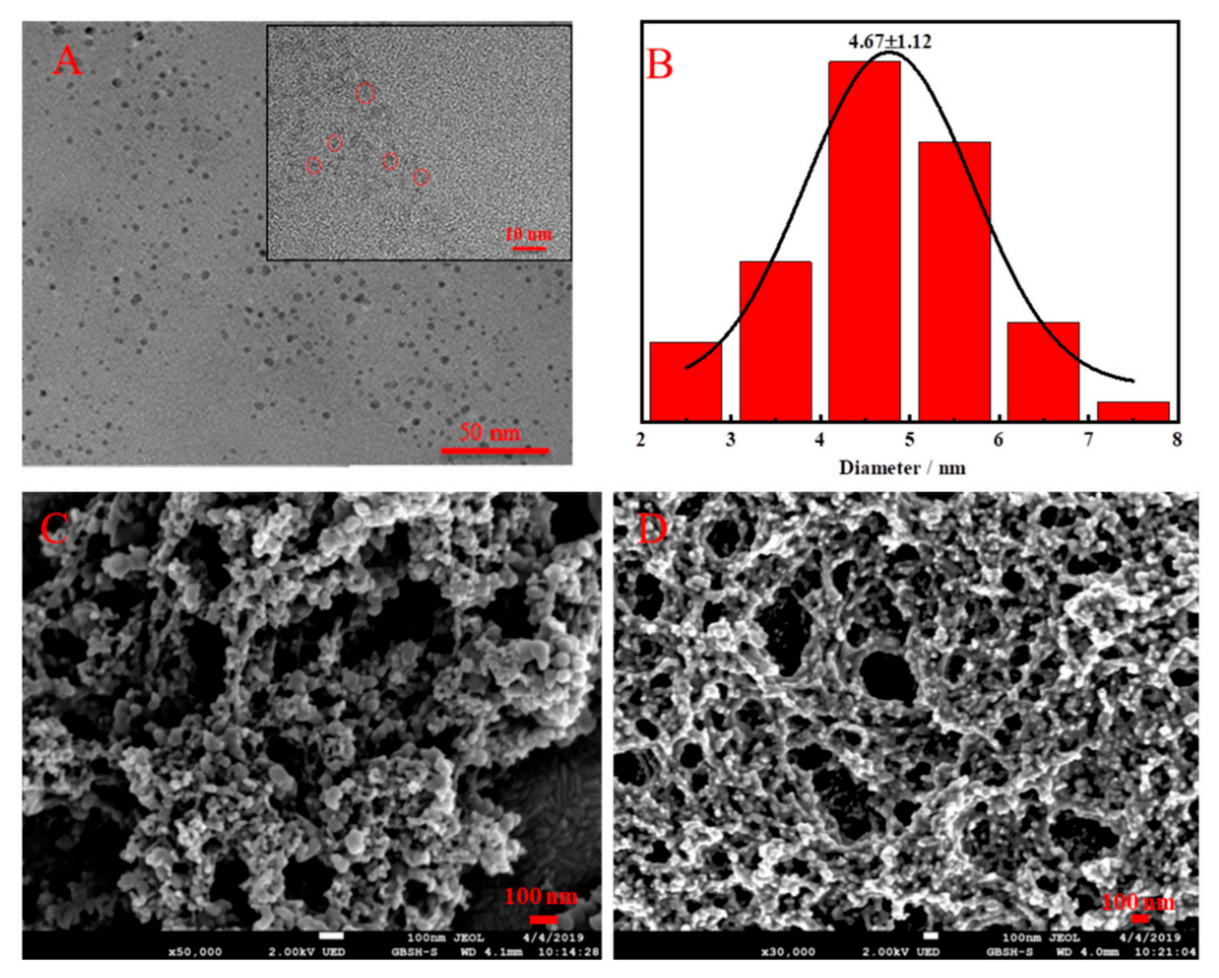
(**A**) A TEM image of the Cu NCs (inset shows an HR-TEM image of the Cu NCs) and (**B**) the diameter distribution of the Cu NCs. (**C**,**D**) are SEM images of Tb-GMP and Cu NCs@Tb-GMP, respectively.

The microstructures, elements and optical properties of the Cu NCs, Tb-GMP and Cu NCs@Tb-GMP were investigated. The morphologies of the Cu NCs, Tb-GMP and Cu NCs@Tb-GMP were characterized by TEM and SEM. Figure 1A and the inset showed that the Cu NCs exhibited a random distribution characteristic of excellent dispersion, and the diameter distribution of the Cu NCs was approximately 4.67 ± 1.12 nm (Figure 1B). SEM images of the ICP and Cu NCs@Tb-GMP are shown in Figure 1C,D, from which some information about the microstructures could be obtained. Tb-GMP was clearly shown to be a spherical colloid. No obvious changes were observed in the microstructure when the Cu NCs were added to Tb-GMP, which proved that the addition of the Cu NCs had no effect on the morphology of Tb-GMP. Thus, a ratiometric fluorescent probe with an ICP network based on Tb and GMP containing Cu NCs was successfully prepared, and the Cu NCs could be used to sensitize Tb-GMP, enhancing its fluorescence intensity. Furthermore, using Cu NCs as an internal standard [36] could effectively correct the error and improve the accuracy of the experiments.

**Figure 2:**
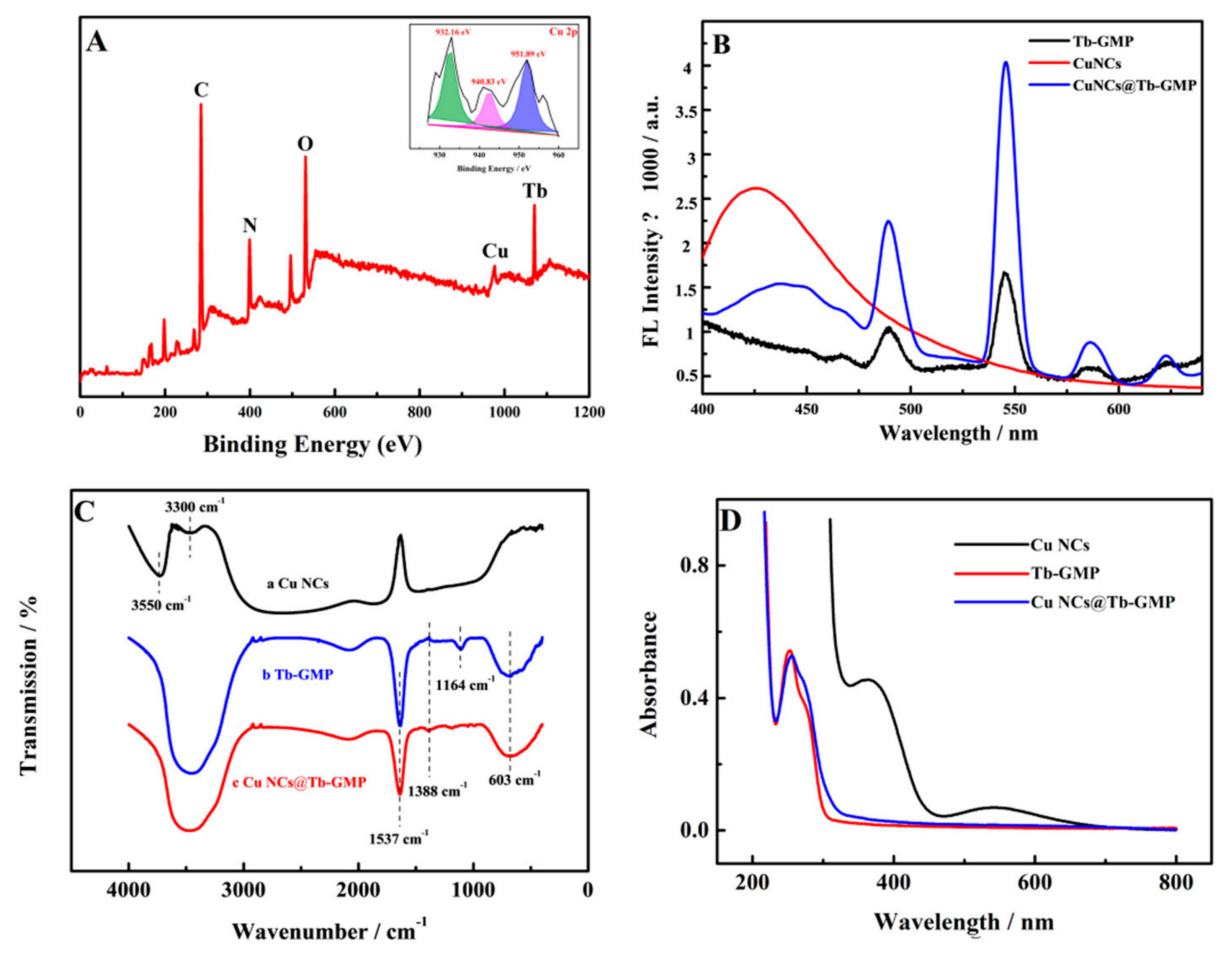
(**A**) The XPS spectrum of Cu NCs@Tb-GMP with the Cu 2p region (inset). (**B**) The fluorescence properties of the Cu NCs (red curve), Tb-GMP (black curve) and Cu NCs@Tb-GMP (blue curve). (**C**) The FT-IR spectra of the Cu NCs (black curve), Tb-GMP (blue curve) and Cu NCs@Tb-GMP (red curve). (**D**) The UV–vis absorption spectra of the Cu NCs (black curve), Tb-GMP (red curve) and Cu NCs@Tb-GMP (blue curve).

XPS was used to explore the elemental compositions of the synthetic materials, as shown in Figure 2A. All the expected elements were present, including C, N, O, Cu and Tb, and their characteristic peaks were located at 285.51, 398.93, 530.97, 950.87 and 1073.23 eV, respectively. Three peaks could be identified in the high-resolution XPS spectrum of Cu (inset). The peaks located at 932.16, 940.83 and 951.89 eV could be assigned to Cu 2p_2/3_ and Cu 2p_1/2_ [30]. The XPS results showed that the Cu NCs@Tb-GMP nanocomposite contained all the elements of the raw materials, which proved that it had been successfully synthesized [16]. The fluorescence properties of the Cu NCs (red curve), Tb-GMP (black curve) and Cu NCs@Tb-GMP (blue curve) at an excitation wavelength of 330 nm are shown in Figure 2B. The red curve shows that the maximum emission wavelength of the Cu NCs was approximately 425 nm under the optimal excitation wavelength of 330 nm. The black curve provided some information indicating that the emission wavelengths of Tb-GMP situated at 489, 545, 587 and 642 nm due to the coordination of O6 and N7 moieties to Tb^3+^ promoted the energy transfer from the guanine base to the emissive D_4_ state of the Tb^3+^ in the process of self-assembly. To verify whether the Cu NCs were coated with Tb-GMP, the blue curve that we obtained was compared with the red and black curves, and no overlap between the emission wavelengths of the Cu NCs and Tb-GMP was observed at the same excitation wavelength, demonstrating that the Cu NCs were successfully encapsulated in the Tb-GMP network.

Figure 2C shows the FT-IR spectra of the Cu NCs (black curve), Tb-GMP (blue curve) and Cu NCs@Tb-GMP (red curve). The characteristic peaks of the Cu NCs at 3550 and 3300 cm^−1^ were attributed to amino groups (NH_2_). However, the peaks were not obvious, indicating that EDA was connected with the Cu core through NH_2_, which was consistent with a previous reference (black curve) [25]. As shown in the blue curve, the strong absorption at 1537 cm^−1^ was attributed to the pyrimidine ring vibration of GMP. The absorption at 603 cm^−1^ was attributed to the nonsymmetrical bending of PO_4_^3+^ in GMP. The weak absorption peak at 1388 cm^−1^ came from the pyrimidine ring of GMP. The peak at 1164 cm^−1^ may have been the result of the vibration characteristics of the GMP sugar ring. FT-IR spectroscopy proved that Tb/GMP was synthesized successfully. In the FT-IR spectrum of Cu NCs@Tb-GMP (red curve), we observed the previously mentioned characteristic peaks. Based on the previous analysis, the Cu NCs@Tb-GMP nanocomposites were successfully synthesized [37]. Figure 2D shows the UV-vis spectra of the Cu NCs (black curve), Tb-GMP (red curve) and Cu NCs@Tb-GMP (blue curve). The black curve had two peaks at 370 nm and 560 nm. The absorption peak at 370 nm was attributed to the presence of NH_2_ in the Cu NCs. The characteristic peak at 560 nm was consistent with the characteristic surface plasmon resonance band of the Cu NCs. The absorption peaks of the red and black curves at 240 nm were caused by the π-π* transition in the nanocomposite, whereas the disappearance of the absorption peak of the black curve indicated that the Cu NCs were successfully encapsulated in Tb-GMP, which further proved the successful synthesis of the composite materials and was consistent with previous research [38].

### 3.2. Optimization Assay

**Figure 3:**
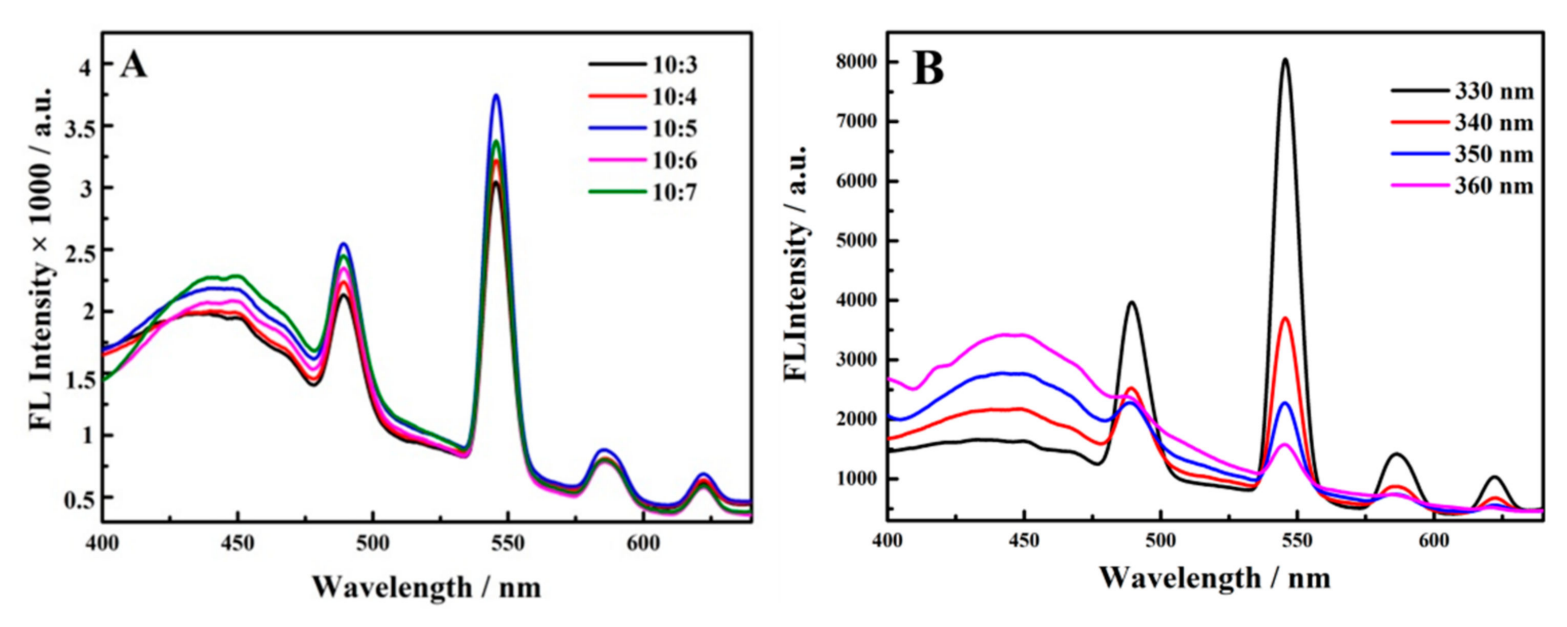
(**A**) The Cu NCs were added to Tb-GMP in different ratios. (**B**) The determination of the optimal excitation wavelength of Cu NCs@Tb-GMP.

To explore the volume ratio of Cu NCs to Tb-GMP in the process of synthesis, a series of experiments were designed. The amount of Tb-GMP remained the same, while the amount of Cu NCs was changed to obtain volume ratios of 10:3, 10:4, 10:5, 10:6 and 10:7. The results are shown in Figure 3A, from which a conclusion could be obtained. When the volume ratio of Tb-GMP to Cu NCs was 10:5, the fluorescence intensity of the Cu NCs@Tb-GMP nanocomposite was the strongest, and the emission peaks of the Cu NCs and Tb-GMP existed simultaneously. Based on the above experiments, 10:5 was chosen as the optimal volume ratio for the synthesis of Cu NCs@Tb-GMP in the subsequent experiments; when the content of Cu NCs was low, they were insufficient to sensitize Tb-GMP, whereas when the content of Cu NCs was too high, the fluorescence characteristics of Tb-GMP were good, with an inconspicuous Cu NCs emission peak. After the synthesis of Tb-GMP, experiments were conducted to determine the optimal excitation wavelength. Excitation wavelengths of 330, 340, 350 and 360 nm were employed in the experiments (Figure 3B), and 330 nm was found to be the optimal excitation wavelength of Tb-GMP.

### 3.3. Linearity of the ALP Ratiometric Fluorescent Sensor in ALP Detection

**Figure 4:**
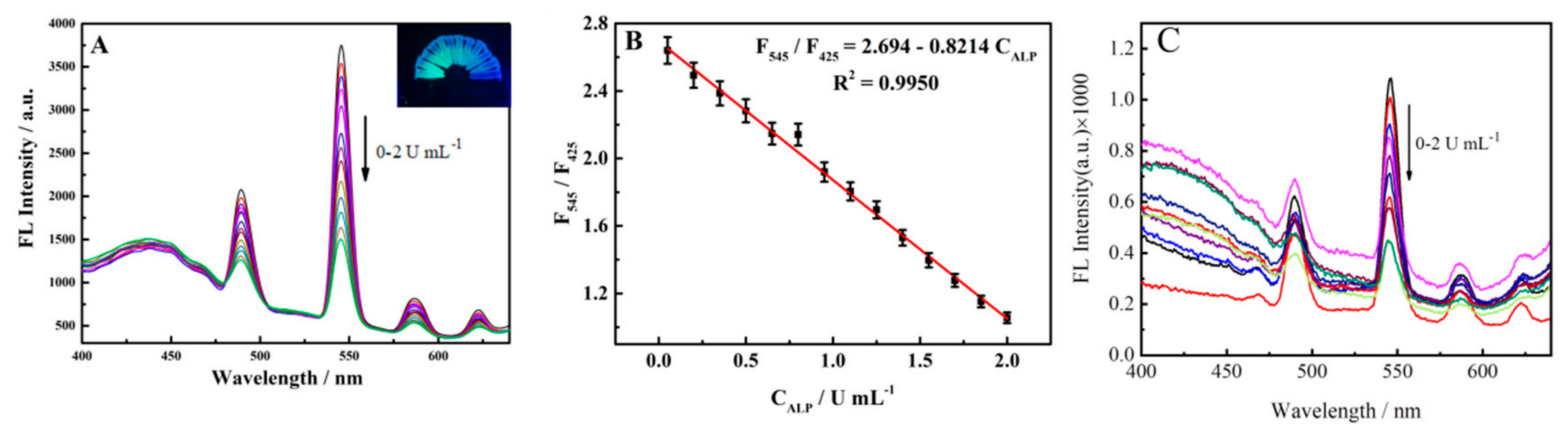
(**A**) The response of the Cu NC@Tb-GMP sensor when exposed to ALP solutions with different concentrations. The inset displays the corresponding images of ALP solutions with different concentrations under 365 nm UV light. (**B**) The plot of the response of the Cu NC@Tb-GMP ratiometric fluorescent sensor as a function of the concentration of ALP. (**C**) The response of the Tb-GMP sensor when exposed to ALP solutions with different concentrations.

Under the optimal experimental conditions, different concentrations of ALP were added to the prepared Cu NCs@Tb-GMP, and the fluorescence response was detected after a 30 min reaction at 37 °C. The mechanism of the ratiometric fluorescent sensor is shown in Scheme 1. For the Cu NCs@Tb-GMP nanocomposites under an excitation wavelength of 330 nm, the emission wavelengths of the nanocomposite were 425 and 545 nm. For Cu NCs@Tb-GMP in the presence of ALP, the phosphate group in GMP could be hydrolyzed, leading to the structural damage of the Cu NCs@Tb-GMP, leading to the wavelength at 545 nm decreasing. Simultaneously, the Cu NCs were released from the Cu NCs@Tb-GMP, while the wavelength at 425 nm had no effect, which may have been due to the surface of Tb-GMP possessing so many Cu NCs that the Cu NCs after being released from Cu NCs@Tb-GMP uninfluenced the fluorescence intensity. The ratio of the two peaks was used to construct a relationship. The results are shown in Figure 4A, from which much information was obtained. The fluorescence intensity of Tb-GMP decreased (545 nm) as the concentration of ALP increased between 0–2 U mL^−1^, whereas the fluorescence intensity of the Cu NCs remained unchanged (425 nm). The inset in Figure 3A displays the corresponding images of ALP solutions with different concentrations under 365 nm UV light (original from the UV flashlight the fixed wavelength). The color of Cu NCs@Tb-GMP became lighter (from left to right), proving that the degree of quenching became stronger with increasing ALP concentration. Figure 4B shows the linear relationship between F_545_/F_425_ and the ALP concentration. The linear equation was F_545_/F_425_ = 2.694 – 0.8214 C_ALP_ and R^2^ = 0.9950, and the detection limit (LOD) was 0.002 U mL^−1^, which suggested that the prepared sensor based on the Cu NCs@Tb-GMP ratiometric fluorescent probe could detect ALP sensitively and accurately. As shown in Figure 4C, the Tb-GMP detects the various concentrations of ALP, and the fluorescence intensity of Tb-GMP was nearly lower than three times that of Cu NCs@Tb-GMP at 545 nm, demonstrating that the Cu NCs possess the ability to sensitize Tb-GMP to improve the intensity. Compared with other types of ALP probes (Table 1), the prepared Cu NCs@Tb-GMP had a lower detection limit and a wider detection range, attesting that the prepared sensor based on the Cu NCs@Tb-GMP ratiometric fluorescent probe had better practicability and could be more effective at detecting ALP in real samples.

### 3.4. Stability, Salt Tolerance, Selectivity and Repeatability of the Probe

**Figure 5:**
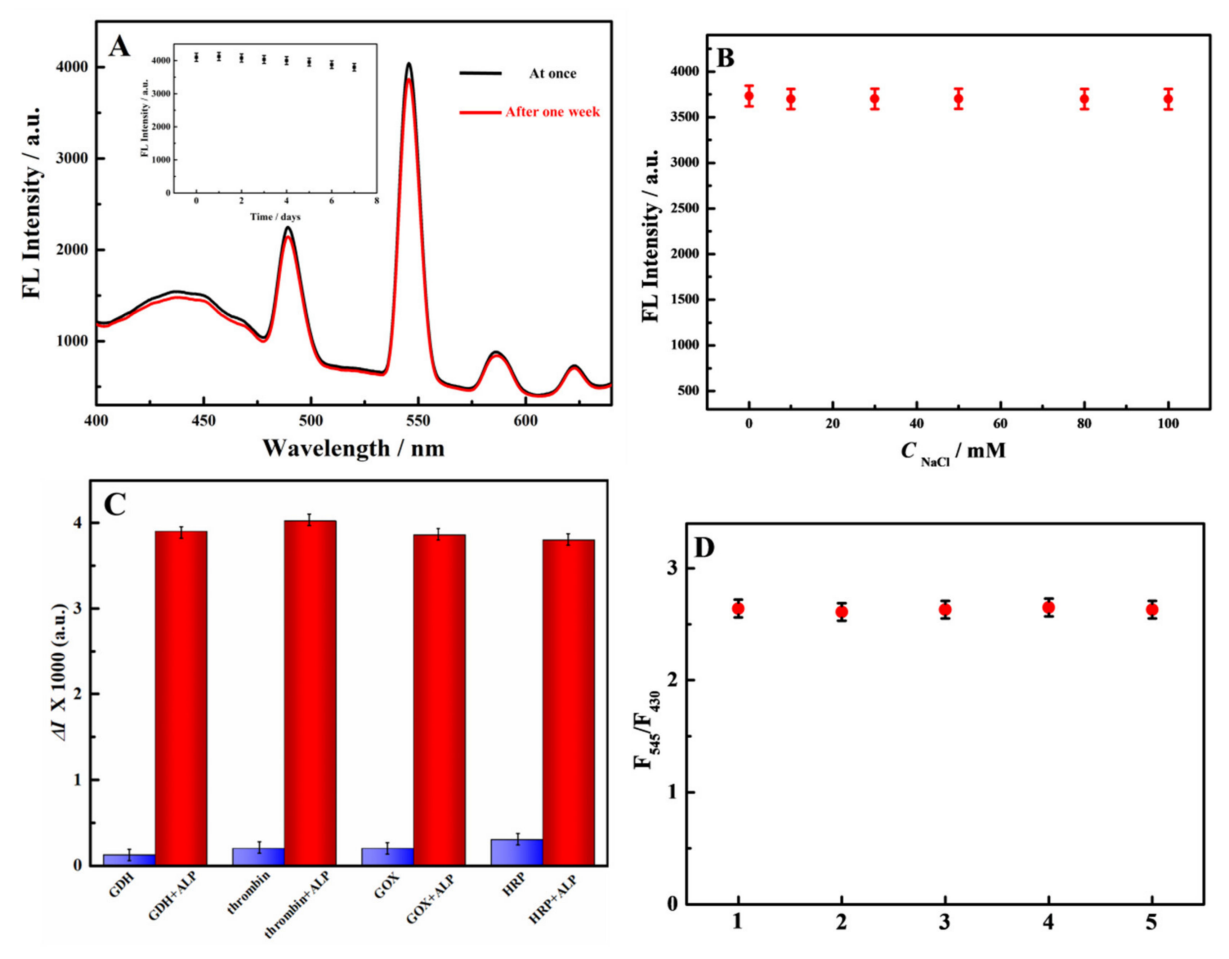
(**A**) The stability of Cu NCs@Tb-GMP after one week; the inset shows the stability each day of the week. (**B**) The salt tolerance of Cu NCs@Tb-GMP. (**C**) The selectivity of other interfering substances over ALP: GDH, thrombin, GOx and HPR. (**D**) The repeatability of Cu NCs@Tb-GMP over five experiments.

Cu NCs possess outstanding stability due to this synthesis method [25]. The Cu NCs could be stable in Tb-GMP. A portion of Cu NCs has been encapsulated into Tb-GMP, and others are located on the surface of Tb-GMP, due to the Cu NCs@Tb-GMP synthesis process. The Cu NCs connect with Tb-GMP were not through any chemical bonds. Therefore, Cu NCs could sustain the stability in the Cu NCs@Tb-GMP. To explore the stability of Cu NCs@Tb-GMP, the synthesized nanocomposite was stored at 4 °C for 7 days, and its fluorescence intensity was detected and recorded at the same excitation wavelength. As shown in Figure 5A, the fluorescence intensity of Cu NCs@Tb-GMP decreased slightly after 7 days but remained basically unchanged. The inset shows the stability of Cu NCs@Tb-GMP over seven days, indicating that the ratiometric fluorescent probe synthesized in the experiment had high stability and would not undergo structural changes for a long time at 4 °C. Meanwhile, experiments were performed to further investigate the salt tolerance of Cu NCs@Tb-GMP, that is, a certain amount of NaCl was added to the synthesized ratiometric fluorescent probe to gradually increase its concentration from 0 to 100 mM, and at the same time, the fluorescence intensity was measured and recorded under a fixed excitation wavelength. As shown in Figure 5B, the fluorescence intensity of Cu NCs@Tb-GMP did not change significantly with increasing NaCl concentration, which indicated that the synthesized Cu NCs@Tb-GMP ratiometric fluorescent probe still had excellent stability in a high concentration salt solution.

In addition, the selectivity of Cu NCs@Tb-GMP for ALP detection was further studied. Other enzymes that may exist in freshwater lakes, such as GDH, thrombin, GOx and HRP, were selected as interference substances to conduct control experiments under the same experimental conditions. The results are shown in Figure 5C. The effect of the interference substance alone on Cu NCs@Tb-GMP was almost negligible, but when the interference substance and ALP were added at the same time, an obvious quenching phenomenon occurred. The results indicated that only the ALP could enable the hydrolysis of the phosphate radical, leading to the structure and energy transmission of Cu NCs@Tb-GMP being damaged. Additionally, the prepared Cu NCs@Tb-GMP had good anti-interference ability during the detection of ALP and could be used for detection in real water samples. Based on this, we selected Cu NCs@Tb-GMP toward ALP over other chosen interference substances.

Moreover, some experiments were designed to verify the reproducibility of Cu NCs@Tb-GMP under the same conditions. The satisfactory results are shown in Figure 5D. In many experiments, the ratio of the fluorescence intensities of the synthesized Cu NCs@Tb-GMP ratiometric fluorescent probe at 545 nm and 425 nm did not vary considerably, which proved that the synthesized probe had good repeatability.

### 3.5. Detection of ALP in Real Samples

Water quality ensures people’s health, and the content of ALP in water is more important than other indicators used to evaluate water quality. Therefore, the accurate and sensitive determination of ALP in water is vital for evaluating water quality. Three samples, which were obtained from a local river (Yantai City), were prepared, and the results are displayed in Table 2. The recovery was between 96.7% and 108.2%, and the relative standard deviation of recovery was obtained by each water sample have three parallel experiments was less than 3%. The experimental results were satisfactory, and it is expected that Cu NCs@Tb-GMP can be applied to ALP detection in various water samples from the environment.

## 4. Conclusions

In this paper, Cu NCs were encapsulated in an ICP based on Tb^3+^ and GMP nanocomposites through a certain reaction to form a new type of ratiometric fluorescent probe called Cu NCs@Tb-GMP. A ratiometric fluorescent sensor strategy for the detection of ALP in water samples was developed based on this ratiometric fluorescent probe. Compared with simple Tb-GMP, Cu NCs@Tb-GMP had much higher fluorescence intensity, and its salt resistance and stability were satisfactory. When ALP was added to Cu NCs@Tb-GMP, the fluorescence intensity corresponding to Tb-GMP showed a good linear relationship with the change in ALP, but the fluorescence corresponding to the Cu NCs basically remained unchanged. This satisfying result proved that the ratiometric fluorescent sensor constructed in this paper had good stability, good reproducibility and high selectivity for ALP and provides a new way to detect water eutrophication.

## Figures and Tables

**Table 1:** A comparison of ALP detection of different kind of fluorescent probes.

Nanoprobes	Linear Range/U mL^−1^	Detection Limit/U mL^−1^	Ref.
coumarin@Tb-GMP ^a^	0.025–0.2	0.01	[39]
AuNPs/GO ^b^	0.1–1	0.009	[40]
ATP-Cu ^c^	0.03–0.3	0.03	[41]
Cu(BCDS ^d^)_2_^2−^	0.027–0.220	0.027	[42]
Cu NCs@Tb-GMP	0.002–2	0.002	This work

^a^ Terbium-guanine monophosphate; ^b^ Gold nanoparticles/graphene oxide; ^c^ Adenosine triphosphate -copper nanozymes; ^d^ Bathocuproine disulfonate.

**Table 2:** The recoveries for ALP determination in samples.

Sample	Add/U mL^−1^	Founded/U mL^−1^	Recovery/%
1	0.2000	0.2164	108.2
	0.5000	0.5036	100.7
2	0.2000	0.2120	106.0
	0.5000	0.4982	99.64
3	0.2000	0.2127	106.4
	0.5000	0.4835	96.70

## Data Availability

Not applicable.
